# Different-Level Simultaneous Minimization Scheme for Fault Tolerance of Redundant Manipulator Aided with Discrete-Time Recurrent Neural Network

**DOI:** 10.3389/fnbot.2017.00050

**Published:** 2017-09-11

**Authors:** Long Jin, Bolin Liao, Mei Liu, Lin Xiao, Dongsheng Guo, Xiaogang Yan

**Affiliations:** ^1^School of Information Science and Engineering, Lanzhou University Lanzhou, China; ^2^College of Information Science and Engineering, Jishou University Jishou, China; ^3^School of Information Science and Engineering, Huaqiao University Xiamen, China; ^4^Department of Computer Science, University of Otago Dunedin, New Zealand

**Keywords:** redundant manipulator, different level, fault tolerance, physical constraint, discrete-time recurrent neural network

## Abstract

By incorporating the physical constraints in joint space, a different-level simultaneous minimization scheme, which takes both the robot kinematics and robot dynamics into account, is presented and investigated for fault-tolerant motion planning of redundant manipulator in this paper. The scheme is reformulated as a quadratic program (QP) with equality and bound constraints, which is then solved by a discrete-time recurrent neural network. Simulative verifications based on a six-link planar redundant robot manipulator substantiate the efficacy and accuracy of the presented acceleration fault-tolerant scheme, the resultant QP and the corresponding discrete-time recurrent neural network.

## 1. Introduction

In recent decades, robotics has drawn more and more attention in scientific areas and engineering applications. Many researches have been focused on this topic, and various kinds of robots have thus been developed and investigated (Roberts and Maciejewski, 1996; Sun et al., 2009; Zhang and Zhang, 2012, 2013; Li et al., 2014; Jin and Zhang, 2015; Jin et al., 2016; Jin and Li, 2017; Jin et al., 2017a,b; Zhang et al., 2017a,b). As a typical kind of robot, redundant manipulators have played a more and more important role in numerous fields of engineering applications (Roberts and Maciejewski, 1996; Zhang and Zhang, 2012, 2013; Jin and Zhang, 2015; Liao and Liu, 2015; Jin et al., 2016, 2017a,b; Jin and Li, 2017; Zhang et al., 2017a,b). For redundant manipulators, they can accomplish subtasks easily and dexterously and optimization of various performance criteria, since they possess more degrees of freedom (DOF) than the minimum amount required to accomplish a given end-effector primary task. Recent progresses have shown advantages of using adaptive neural networks for the control of nonlinear systems (Tang et al., 2014, 2017). For example, an adaptive control scheme was provided in Tang et al. (2014) for robot manipulator systems with unknown functions and dead-zone input by using adaptive neural networks, of which the parameters of the dead zone are assumed to be unknown but bounded. One of the fundamental issues in operating such a redundant manipulator is the inverse-kinematics problem (or termed, redundancy-resolution problem). Specifically, the joint trajectories need to be generated online based on the provided desired Cartesian trajectories of the end-effector. That is, if the manipulator control scheme is fault tolerant, the end-effector can fulfill the required task even if its joint fails. As one essential and challenging issue in inverse kinematics, it is important to tolerate joint failure online during the task execution. For example, in the remote applications such as space or sea exploration, repairing broken actuators is costly or even impossible and the probability of their failure is increased due to the hostile operating environment. To say the least, it may induce the damage to people and/or manipulator if a manipulator without a fault-tolerant scheme being equipped suddenly encounters a joint-lock situation during the execution. Thus, the fault-tolerant ability is an important or even indispensable design criterion for robotic systems.

More recently, the fault tolerance has attracted significant interest in the societies of academia and industry because of the increased demands in practical applications for safety and productivity as well as operating efficiency. Numerous researches on the topic of fault tolerance have thus been presented and investigated (Roberts and Maciejewski, 1996; Izumikawa et al., 2005; Siqueira and Terra, 2009; Li and Zhang, 2012). Authors in Siqueira and Terra (2009) described the fault occurrence for a three-link manipulator based on a Markovian jump model, which took into account all possible fault sequences in a three-link manipulator robot, and defined guidelines to control an *n*-link manipulator robot with several faults. Izumikawa et al. (2005) designed a controller of a fault-tolerant system with a signal observer for a strain gauge sensor fault. By switching from the controller for normal mode to the controller for unnormal mode, the stability and the control performance of such a system were maintained. Generally speaking, the existing methods for fault tolerance can be categorized as the passive type and the active type (Zhang and Jiang, 2008). The former one fixes and designs the corresponding controllers to be robust against the presumed faults, which needs neither fault detection nor controller reconfiguration, with limited capabilities for fault tolerance. The latter one reacts to the failures of the system actively by reconfiguring control actions to maintain the stability and acceptable performance of the entire system (Bustan et al., 2014).

A fault-tolerant scheme was presented by adding a matrix-vector form equality constraint for the faulty joint, which took the limits of joint angle and joint velocity into consideration (Li and Zhang, 2012). However, this fault-tolerant scheme can not handle the joint-acceleration limits. More seriously, this scheme may introduce the undesirable discontinuity phenomenon in the velocity solution because it was investigated at the joint-velocity level. Thus, it is worthy to investigate the scheme for fault-tolerance of redundant robot manipulators at the joint-acceleration level, which can effectively remedy the discontinuity phenomenon at the joint-velocity level and incorporates robot dynamic. However, up to now there is almost no study on the fault tolerance of redundant robot manipulators on the combination of different level. In other words, the study on the fault tolerance of redundant robot manipulators at the different level is still rare despite its appealingness.

In this paper, we make progress along this direction by presenting a different-level simultaneous minimization scheme, which takes both the robot kinematics and robot dynamics into account. The scheme is then reformulated as a quadratic program (QP) subject to equality and bound constraints. In order to solve such a QP problem, a discrete-time recurrent neural network is developed and applied to online solution of the QP problem. Simulative results based on a six-link planar robot manipulator further illustrate the efficacy and superiority of the proposed fault-tolerant scheme for fault tolerance of redundant robot manipulators.

## 2. Preliminaries and scheme formulation

To lay a basis for further discussion, the relationship between the end-effector velocity *ṙ* ∈ *R*^*m*^ and the joint velocity $\dot{q}\in R^{n}$ for redundant robot manipulators can be given hereinafter directly (Zhang and Zhang, 2012)

(1) $$\begin{array}{l} J(q)\dot{q}=\dot{r}, \end{array}$$

where *J*(*q*) ∈ *R*^*m*×*n*^ is the Jacobian matrix of the end-effector with *q* being the joint-angle vector. By differentiating Equation (1) with respect to time *t*, the relationship between the end-effector acceleration $\ddot{r}$ and the joint acceleration $\ddot{q}$ is obtained as follows (Zhang and Zhang, 2012):

(2) $$\begin{array}{l} J(q)\ddot{q}={\ddot{r}}_{\text{b}}=\ddot{r}-\dot{J}(q)\dot{q}, \end{array}$$

where $\dot{J}\left(q\right)$ is the time derivative of *J*(*q*). Note that, since the manipulator system is redundant (i.e., *m* < *n*), Equations (1) and (2) are under-determined, and generally admit an infinite number of feasible solutions in terms of inverse kinematics. This implies the ability to accommodate more functional constraints such as fault tolerance. For example, once a joint is stuck, other joints can take over its workload and move the end-effector to its goal via a fault-tolerant scheme. It is worth mentioning here that the fault-tolerant scheme investigated in Li and Zhang (2012) was based on Equation (1) (i.e., at the joint-velocity level), while the fault-tolerant scheme presented in the ensuing subsections is based on Equation (2) (i.e., at the joint-acceleration level).

For the online solution of Equation (2), the following QP-oriented formulation can be used (Zhang and Zhang, 2012):

(3) $$\begin{array}{l} \text{minimize}\;\;\;\;\;{\ddot{q}}^{\text{T}}\Lambda \ddot{q}/2+c^{\text{T}}\ddot{q}, \end{array}$$

(4) $$\begin{array}{l} \text{subject}\;\text{to}\;\;\;\;J(q)\ddot{q}={\ddot{r}}_{\text{b}}, \end{array}$$

where coefficients Λ ∈ *R*^*n*×*n*^ and *c* ∈ *R*^*n*^ are defined accordingly for a specific redundancy-resolution scheme. In addition, superscript T denotes the transpose of a matrix or vector.

However, without appropriate remedied measures, when a manipulator suffers a joint failure, its performance would be significantly affected, and even worse, the manipulator fails to complete the prescribed path task. In safety-critical systems, the consequences of a minor fault in a system component can be catastrophic. Therefore, the demands on reliability, safety and fault tolerance are generally high. It is necessary to take fault tolerance into consideration in the above inverse-kinematic scheme (i.e., Equations 3 and 4) in order to improve the reliability and availability while tracking a desirable path. Inspired by Roberts and Maciejewski (1996), at a time instant, if there are *n*_f_ joints being locked (e.g., the *i*th, …, and *j*th joints with *i*, *j* ∈ [1, 2, …, *n*] and *i* ≠ *j*), then we directly remove the corresponding joint-acceleration variables in the scheme. For example, if a failed joint *i* is locked, the corresponding relationship between the end-effector acceleration and the joint acceleration is obtained as

(5) $$\begin{array}{l} {[}^{i}J(q)]{[}^{i}\ddot{q}]={\ddot{r}}_{\text{b}}=\ddot{r}-{[}^{i}J\dot{(}q)]{[}^{i}\dot{q}], \end{array}$$

where ${\;}^{i}J\left(q\right)=\left[j_{1},\cdots \;,j_{i-1},j_{i+1},\cdots \;,j_{n}\right]$ and ${\;}^{i}\dot{J}\left(q\right)=\left[{\dot{j}}_{1},\cdots \;,{\dot{j}}_{i-1},{\dot{j}}_{i+1},\cdots \;,{\dot{j}}_{n}\right]$ with *j*_*h*_ and ${\dot{j}}_{h}$ denoting the *h*th column of *J*(*q*) and $\dot{J}\left(q\right)$, respectively. In addition, ${\;}^{i}\dot{q}=\left[{\dot{q}}_{1},\cdots \;,{\dot{q}}_{i-1},{\dot{q}}_{i+1},\cdots \;,{\dot{q}}_{n}\right]$ and ${\;}^{i}\ddot{q}=\left[{\ddot{q}}_{1},\cdots \;,{\ddot{q}}_{i-1},{\ddot{q}}_{i+1},\cdots \;,{\ddot{q}}_{n}\right]$. The reduced Equation (5) then determines the kinematic properties of the degraded system.

By incorporating the joint physical constraints as well as the robot dynamic presented in Appendix (Supplementary Material), the different-level simultaneous minimization scheme for fault tolerance of robot manipulators is written as

(6) $$\begin{array}{l} \text{minimize}\;\alpha ({\ddot{\theta }}^{\text{T}}\text{W}\ddot{\theta }\text{/2}+p^{\text{T}}\ddot{\theta })+\beta \tau^{\text{T}}\text{T}\tau /2, \end{array}$$

(7) $$\begin{array}{l} \text{subject}\;\text{to}\;J(\theta )\ddot{\theta }={\ddot{r}}_{\text{a}}, \end{array}$$

(8) $$\begin{array}{l} \tau =H\ddot{\theta }+c_{\tau }(\dot{\theta },\theta )+g_{\tau }(\theta ), \end{array}$$

(9) $$\begin{array}{l} \theta^{-}⩽\theta ⩽\theta^{+}, \end{array}$$

(10) $$\begin{array}{l} {\dot{\theta }}^{-}⩽\dot{\theta }⩽{\dot{\theta }}^{+}, \end{array}$$

(11) $$\begin{array}{l} {\ddot{\theta }}^{-}⩽\ddot{\theta }⩽{\ddot{\theta }}^{+}, \end{array}$$

(12) $$\begin{array}{l} \tau^{-}⩽\tau ⩽\tau^{+}, \end{array}$$

where α ∈ (0, 1) and β ∈ (0, 1) are the weighting factors with α+β = 1 numerically; θ, $\dot{\theta }$, $\ddot{\theta }$ and τ denote the *n*_r_ dimensional joint-angle, joint-velocity, joint-acceleration and joint-torque vectors, respectively; $W=I\in R^{n_{\text{r}}\times n_{\text{r}}}$, and $J\left(\theta \right)\in R^{m\times n_{\text{r}}}$, $p\in R^{n_{\text{r}}}$; $b={\ddot{r}}_{\text{a}}+K_{\text{v}}\left({ṙ}_{\text{d}}-J\left(\theta \right)\dot{\theta }\right)+K_{\text{p}}\left(r_{\text{d}}-f\left(\theta \right)\right)\in R^{m}$; ${\ddot{r}}_{\text{a}}=\ddot{r}-\dot{J}\left(\theta \right)\dot{\theta }$ with $\dot{J}\left(\theta \right)\in R^{m\times n_{\text{r}}}$ and *n*_r_ = *n* − *n*_f_ [*n*_f_ denotes the number of faulty joint(s)]. In addition, *H* denotes the *n*_r_ × *n*_r_ dimensional inertia matrix; *c*_τ_ denotes the *n*_r_ dimensional Coriolis/centrifugal force vector and *g*_τ_ denotes the *n*_r_ dimensional gravitational force vector. Besides, $\tau^{\pm }=\pm 140\times 1_{v}$ N·m. For simplicity and for example, α is set to be 0.995 (i.e., β = 0.005) in the ensuing simulations.

Remark 1: Fault detection is a fundamental, specialized and relatively independent part for fault tolerance, for which many methods have been proposed. These methods can be classified into two categories: model-based methods and data-based methods (Yüksel and Sezgin, 2010). Model-based methods obtain the deviations signals between the model and the real system named as residuals to detect faults. Data-based methods are based on only processing input and output signals of the system to detect faults. In this paper, for focusing on the superior performance of the fault-tolerant scheme in faulty situation, it can be simply assumed that the fault detection/diagnosis system can always detect and diagnose an unexpected joint fault and immediately give the feedback to the fault-tolerant system. Once the fault-tolerant system receives such a feedback, it activates the reconfiguration mechanism and removes the corresponding joint-acceleration variables in the scheme.

Remark 2: Note that the model disturbance and computational round-off errors always exist in practical application. In order to improve the accuracy of the scheme, the feedback control needs to be incorporated into the forward kinematics equation. One effective approach is to add feedbacks of Cartesian position and velocity error, i.e., instead of using $J\left(\theta \right)\ddot{\theta }={\ddot{r}}_{\text{a}}$, Equation (7) can be replaced with

$$\begin{array}{l} J(\theta )\ddot{\theta }={\ddot{r}}_{\text{a}}+K_{\text{v}}({\dot{r}}_{\text{d}}-J(\theta )\dot{\theta })+K_{\text{p}}(r_{\text{d}}-f(\theta )), \end{array}$$

where *K*_p_ and *K*_v_ are positive-definite symmetric *m* × *m* gain matrices for position-error and velocity-error feedbacks, respectively. In the ensuing simulations and experiments, the diagonal elements of the gain matrices *K*_p_ and *K*_v_ are set as 10 and the off-diagonal elements are set as 0 for simplicity and clarity.

With the aid of conversion techniques given in Cheng et al. (1994), Cheng et al. (1995), and Park et al. (1998), the new bound constraint can thus be written as $\eta^{-}\leq \ddot{\theta }\leq \eta^{+}$, where the *i*th elements of η^−^ and η^+^ are defined respectively as

$$\begin{array}{l} \eta_{i}^{-}=\max\{\gamma_{\text{p}}(\theta_{i}^{-}+\vartheta_{i}-\theta_{i}),\gamma_{\text{v}}({\dot{\theta }}_{i}^{-}-{\dot{\theta }}_{i}),{\ddot{\theta }}_{i}^{-}\}, \end{array}$$

$$\begin{array}{l} \eta_{i}^{+}=\min\{\gamma_{\text{p}}(\theta_{i}^{+}-\vartheta_{i}-\theta_{i}),\gamma_{\text{v}}({\dot{\theta }}_{i}^{+}-{\dot{\theta }}_{i}),{\ddot{\theta }}_{i}^{+}\}, \end{array}$$

where ϑ > 0 is a small constant vector used to scale the safety region. Besides, coefficients γ_p_ > 0 and γ_v_ > 0 determine the deceleration magnitude.

Based on the above analysis, the proposed scheme for physically-constrained redundant robot manipulators can be reformulated into the following standard QP in terms of $\ddot{\theta }$:

(13) $$\begin{array}{l} \text{minimize}\;\alpha ({\ddot{\theta }}^{\text{T}}W\ddot{\theta }/2+p^{\text{T}}\ddot{\theta })+\beta \tau^{\text{T}}\tau /2 \end{array}$$

(14) $$\begin{array}{l} \text{subject}\;\text{to }A\ddot{\theta }=b, \end{array}$$

(15) $$\begin{array}{l} \eta^{-}⩽\ddot{\theta }⩽\eta^{+}, \end{array}$$

where $W=I\in R^{n_{\text{r}}\times n_{\text{r}}}$, $A=J\left(\theta \right)\in R^{m\times n_{\text{r}}}$, $b={\ddot{r}}_{\text{a}}+K_{\text{v}}\left({ṙ}_{\text{d}}-J\left(\theta \right)\dot{\theta }\right)+K_{\text{p}}\left(r_{\text{d}}-f\left(\theta \right)\right)\in R^{m}$, $\tau =H\ddot{\theta }+c_{\tau }\left(\dot{\theta },\theta \right)+g_{\tau }\left(\theta \right)$, and $p=\left(\mu +\nu \right)\dot{\theta }+\mu \nu \left(\theta -\theta \left(0\right)\right)\in R^{n_{\text{r}}}$ with μ > 0 and ν > 0. In addition, $\ddot{\theta }$ and η^±^ are defined the same as before.

Neural networks have been recognized as a powerful tool for real-time processing and successfully applied widely in various control systems (Wang et al., 2015, 2016). In particular, we use the following discrete-time recurrent neural network for solving online the QP problem (Xiao and Zhang, 2013; Zhang and Zhang, 2013).

(16) $$\begin{array}{l} u^{k+1}=u^{k}-\frac{\|e(u^{k}){\|}_{2}^{2}}{\|(M^{T}+I)e(u^{k}){\|}_{2}^{2}}(M^{T}+I)e(u^{k}), \end{array}$$

where || · ||_2_ denotes the two-norm of a matrix; the decision variable vector *u* and its upper and lower bounds *u*^±^ ∈ *R*^*N*^ (with *N* = *n*_r_ + *m*) are defined respectively as

$$\begin{array}{l} u=\left[\begin{array}{c} \ddot{\theta }\\ y \end{array}\right],u^{+}=\left[\begin{array}{c} \eta^{+}\\ \varpi 1_{\text{v}} \end{array}\right],u^{-}=\left[\begin{array}{c} \eta^{-}\\ -\varpi 1_{\text{v}} \end{array}\right], \end{array}$$

with *y* ∈ *R*^*m*^ being the auxiliary decision vector (i.e., dual decision vector) defined corresponding to equality constraint (Equation 14), $1_{\text{v}}={\left[1,\ldots ,1\right]}^{\text{T}}$ denoting an appropriately dimensioned vector composed of ones, constant ϖ = 10^10^ ∈ *R* being defined to replace +∞ numerically, and the augmented matrix *M* and vector *z* being defined respectively as

$$\begin{array}{l} M=\left[\begin{array}{cc} \alpha W+\beta H & -A^{\text{T}}\\ A & 0 \end{array}\right]\in R^{N\times N},\;\;z=\left[\begin{array}{c} p+c_{\tau }\\ -b \end{array}\right]\in R^{N}. \end{array}$$

Besides, $P_{\Omega }\left(\cdot \right):R^{N}\to \Omega$ is a piecewise-linear projection operator defined from space *R*^*N*^ onto set Ω, and the *i*th element of *P*_Ω_(*u*) is defined as

$$\begin{array}{l} {}[P_{\Omega }(u)]i=\{\begin{array}{ll} u_{i}^{-}, & \text{if}\;u_{i}\;⩽\;u_{i}^{-},\\ u_{i}, & \text{if}\;u_{i}^{-}<u_{i}<u_{i}^{+},\\ u_{i}^{+}, & \text{if}\;u_{i}⩾u_{i}^{+}, \end{array}\;\forall i\in \{1,2,\cdots ,N\}. \end{array}$$

## 3. Simulative results

In this paper, a six-link planar manipulator with motor-driven push-rod type joints is presented as a simulative platform to illustrate the effectiveness of the scheme. The hardware system of the six-link planar manipulator, which has six joints, is presented in Zhang and Zhang (2013). The physical parameters of the six-link planar manipulator are shown in Table 1, of which $\theta_{i}^{+}$ and $\theta_{i}^{-}$ denote respectively the upper and lower limits of the *i*th joint-angle vector and *l*_*i*_ denotes the length of the *i*th link, *i* = 1, 2, …, 6. Besides, in the simulations and the ensuing experiment, the final error tolerance of ||*e*(*u*^*k*^)|| is set as 10^−5^ for the discrete-time QP solver Equation (16) with the sampling gap being 0.01 s. The end-effector of the six-link planar redundant robot manipulator is expected to track a square-path with side-length being 0.039 m. In addition, the duration of the path-tracking task is 20 s, ϑ = 0.1 rad, joint-velocity limits ${\dot{\theta }}^{\pm }=\pm 1.5\times 1_{v}$ rad/s, joint-acceleration limits ${\ddot{\theta }}^{\pm }=\pm 2\times 1_{v}$ rad/s^2^ and μ = ν = 4.

**Table 1:** Physical limits of the six-link robot manipulator.

***i***	**$\theta_{i}^{-}$ (rad)**	**$\theta_{i}^{+}$ (rad)**	***l*_*i*_ (m)**	***a*_*i*_ (m)**	***b*_*i*_ (m)**
1	−1.536	1.431	0.301	–	–
2	0.052	0.816	0.290	0.250	0.080
3	0.035	0.621	0.230	0.250	0.080
4	0.052	0.599	0.225	0.190	0.080
5	0.035	0.599	0.214	0.185	0.080
6	0.009	0.445	0.103	0.174	0.080

**Figure 1:**
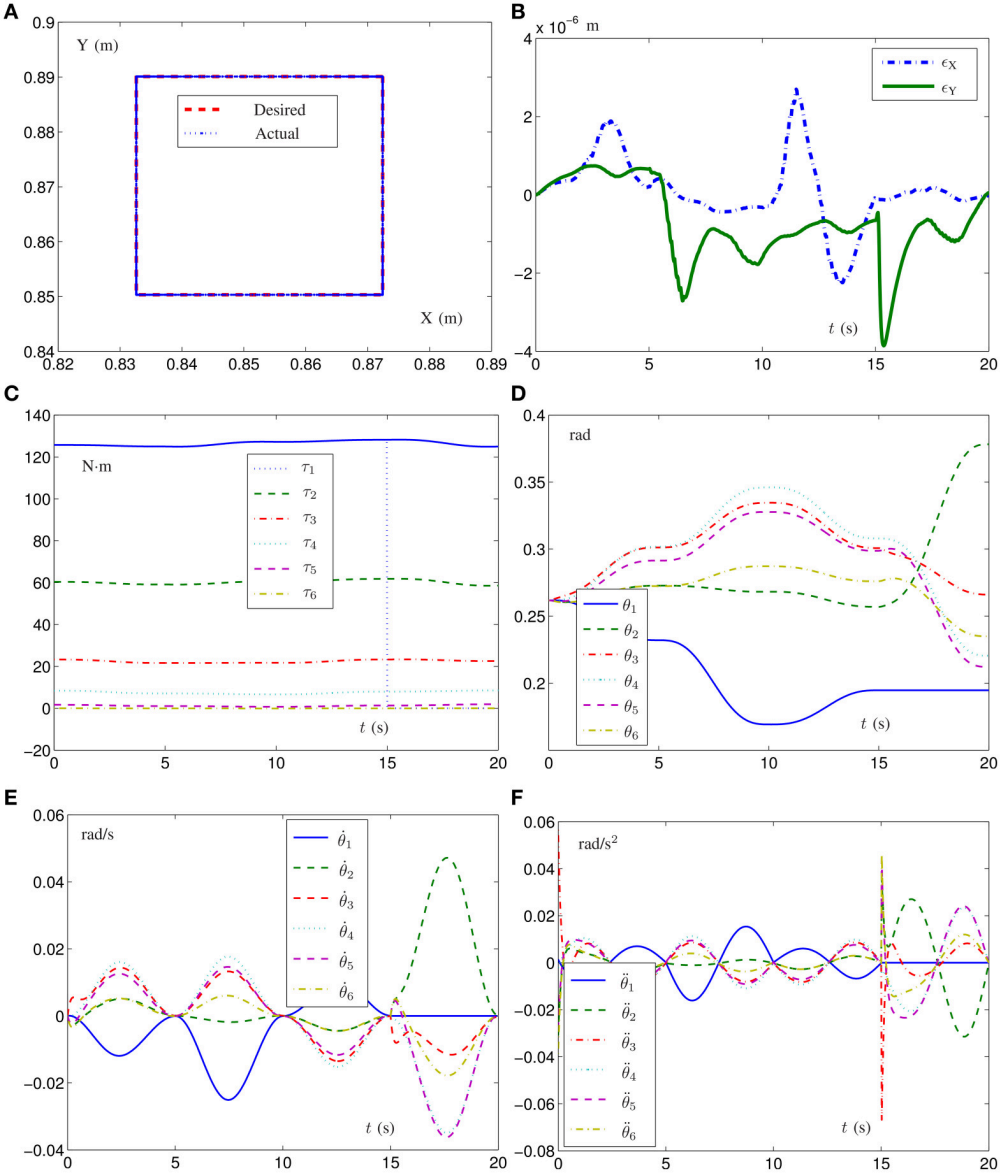
Simulation results of the six-link planar redundant robot manipulator with its end-effector tracking the given square path synthesized by different-level simultaneous minimization scheme (Equations 6–12) and with the first joint being faulty from on *t* = 15 s. **(A)** Desired square-path and actual end-effector trajectory. **(B)** Corresponding tracking position-error profiles. **(C)** Joint-torque profiles. **(D)** Joint-angle profiles. **(E)** Joint-velocity profiles. **(F)** Joint-acceleration profiles.

For comparison and for illustrating the efficacy of the different-level simultaneous minimization scheme (Equations 6–12) in the faulty situation, the simulation results synthesized by scheme (Equations 6–12) with the first joint being faulty from on *t* = 15 s are shown in Figure 1. As observed from Figure 1A, the end-effector motion trajectory is close enough to the desired square path (i.e., with the robot dynamics taken into account, the tracking task is also completed), which substantiates the effectiveness of the different-level simultaneous minimization scheme (Equations 6–12) in the faulty situation. In addition, the tracking position error with the maximal position error being less than 4 × 10^−6^ m shown in Figure 1B further shows the efficacy and accuracy of such a different-level simultaneous minimization scheme. Besides, in Figure 1C, for the first joint torque (i.e., τ_1_ denoted by the blue lines), the solid lines and dashed lines respectively denote the joint-torque profiles corresponding to the no-fault situation and fault-tolerant situation with the first joint being faulty from on *t* = 15 s. As observed from Figure 1C, after *t* = 15 s, the value of the first joint torque becomes zero. With the first joint being faulty from on *t* = 15 s, the values of the joint velocities and joint accelerations are zero, which implies the efficacy of the different-level simultaneous minimization scheme (Equations 6–12) in the faulty situation. In summary, the above simulation results substantiate the efficacy and accuracy of the the different-level simultaneous minimization scheme (Equations 6–12), which takes both the robot kinematics and robot dynamics into account.

**Figure 2:**
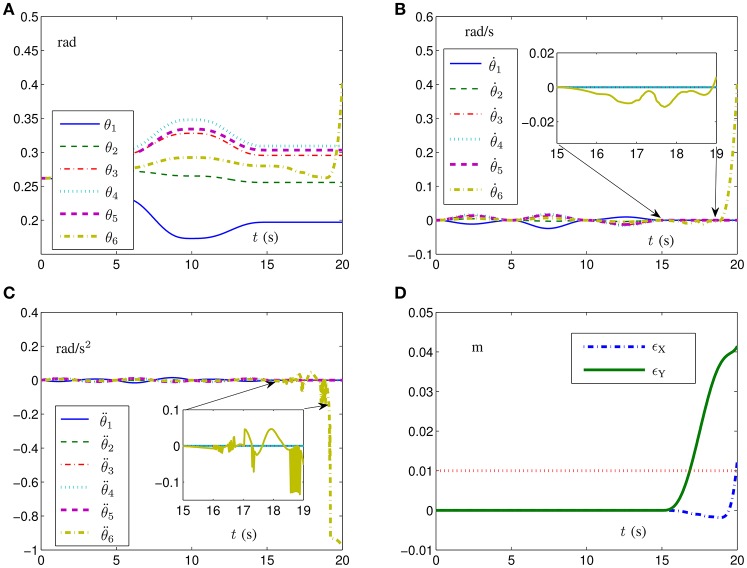
Simulation results of the six-link planar redundant robot manipulator with its end-effector tracking the given square path synthesized by different-level simultaneous minimization scheme (Equations 6–12) with the first five joints being faulty from on *t* = 15 s. **(A)** Joint-angle profiles. **(B)** Joint-velocity profiles. **(C)** Joint-acceleration profiles. **(D)** Corresponding tracking position-error profiles.

Remark 3: Note that, for a fault-tolerant task, it can be classified into the following two cases. (i) The equality constraint is always satisfied; e.g., once some joints are simultaneously faulty, the remainder joints can take over the workload and move the end-effector to its goal via a fault-tolerant scheme. (ii) The equality constraint can not be always satisfied; e.g., with some joints being faulty, the equality constraint does not hold at some time instants, and thus the path-tracking task can not be fulfilled in this situation. Theoretically speaking, the equality constraint should be satisfied all the time. However, strictly speaking, the equality constraint can not be satisfied exactly even for the first case. Specifically, the tracking position-error profiles synthesized by the different-level simultaneous minimization scheme (Equations 6–12) are numerically near zero but nonzero (i.e., 10^−6^). That is because the simulation and computation are performed on a finite-arithmetic finite-memory digital computer. Then, the tracking position error may be inevitable between the desired path and actual trajectory, which is used to be the input of the feedback to track the task in a more accurate manner. To show clearly the second case (i.e., the equality constraint is not always satisfied), the corresponding motion trajectories and tracking errors with the first five joints being locked from on *t* = 15 s are visualized in Figure 2. Specifically, as seen from Figure 2, with the first five joints being faulty from on *t* = 15 s, the values of the corresponding joint velocities and joint accelerations are zero and the corresponding joint angles remain the same as θ(*t* = 15). To distinguish those two types of position error (i.e., the usual computational error, and the failure error due to the lack of feasible solution) as well as to keep the robotic system more reliable, a criterion can be added to the scheme. For example, the emergency brake of the system can be activated for the position error larger than 0.01 m with an increasing trend. As shown in Figure 2D, with the red dotted line denoting the criterion, the robotic system can be stopped at time instant *t* ≈ 16 s to prevent the potential damage(s) to nearby people and/or robot arms.

It is worth pointing out that, although the investigations are based on the joint-lock situation, the efficacy of the proposed different-level simultaneous minimization scheme (Equations 6–12) as well as the resultant QP is not limited to the joint-lock situation. The joint-lock situation is just a representative of lots of joint faulty situations, such as the failure of one joint actuator. In addition, being the representative, the joint-lock situation extensively exists in types of joints (e.g., rotational joints and translational joints). For example, the joints may be locked with a greater probability when the robot works with the sludge. Thus, the proposed different-level simultaneous minimization scheme (Equations 6–12) is generally applicable, and the feasibility and efficacy of such a proposed scheme are not limited by the specific robot structure and failure mode.

## 4. Conclusions

In this paper, by incorporating the physical constraints in joint space, a different-level simultaneous minimization scheme, which takes both the robot kinematics and robot dynamics into account, has been presented and investigated for fault-tolerant motion planning of redundant manipulator in this paper. Then, the scheme has been reformulated as a quadratic program (QP) with equality and bound constraints, which has been solved by a discrete-time recurrent neural network. Simulative verifications based on a six-link planar redundant robot manipulator have substantiated the efficacy and accuracy of the presented acceleration fault-tolerant scheme, the resultant QP and the corresponding discrete-time recurrent neural network.

## Author contributions

LJ and BL presented the scheme. BL and ML designed experiments. LX and DG carried out experiments. XY analyzed experimental results. LJ and BL wrote the manuscript.

### Conflict of interest statement

The authors declare that the research was conducted in the absence of any commercial or financial relationships that could be construed as a potential conflict of interest. The reviewer YZ and handling Editor declared their shared affiliation.
