# Supplementary material for: Different-Level Simultaneous Minimization Scheme for Fault Tolerance of Redundant Manipulator Aided with Discrete-Time Recurrent Neural Network
Source: Front Neurorobot. 2017 Sep 11;11:50. doi: 10.3389/fnbot.2017.00050 (PMC5601992; doi:10.3389/fnbot.2017.00050)
Supplement: Supplementary file 1 [file Presentation1.pdf]

Appendix A
Dynamic Modeling of Planar Robot Manipulator

For the dynamic modeling of the six-link planar robot manipulator presented in [1], it is known from [2], [3] that the dynamic equation of the robot manipulator can be given as

$$\tau = H\ddot{\theta} + c_\tau(\dot{\theta}, \theta) + g_\tau(\theta), \qquad (1)$$

where θ, $\dot{\theta}$, $\ddot{\theta}$ and τ denote the n-dimensional joint-angle, joint-velocity, joint-acceleration and joint-torque vectors, respectively; H denotes the $n \times n$ dimensional inertia matrix; c_τ denotes the n-dimensional Coriolis/centrifugal force vector and g_τ denotes the n-dimensional gravitational force vector. By defining

$$H = \begin{bmatrix} H_{11} & H_{12} & H_{13} & H_{14} & H_{15} & H_{16} \\ H_{21} & H_{22} & H_{23} & H_{24} & H_{25} & H_{26} \\ H_{31} & H_{32} & H_{33} & H_{34} & H_{35} & H_{36} \\ H_{41} & H_{42} & H_{43} & H_{44} & H_{45} & H_{46} \\ H_{51} & H_{52} & H_{53} & H_{54} & H_{55} & H_{56} \\ H_{61} & H_{62} & H_{63} & H_{64} & H_{65} & H_{66} \end{bmatrix},$$

$$c_\tau = [c_{\tau 1} \quad c_{\tau 2} \quad c_{\tau 3} \quad c_{\tau 4} \quad c_{\tau 5} \quad c_{\tau 6}]^{\mathrm{T}},$$

$$g_\tau = [g_{\tau 1} \quad g_{\tau 2} \quad g_{\tau 3} \quad g_{\tau 4} \quad g_{\tau 5} \quad g_{\tau 6}]^{\mathrm{T}},$$

and by using the recursive Newton-Euler algorithm [2], [3], the detailed expressions of elements of matrix H as well as vectors c_τ and g_τ are derived and presented as below.

$H_{11} = H_{21} + (\sum_{i=1}^{6} m_i)l_1(l_1) + (\sum_{i=2}^{6} m_i)l_1l_2c_2 + (\sum_{i=3}^{6} m_i)l_1l_3c_{23} + (\sum_{i=4}^{6} m_i)l_1l_4c_{234} + (m_5+m_6)l_1l_5c_{2345} + m_6l_1l_6c_{23456}$,
$H_{12} = H_{22} + (\sum_{i=2}^{6} m_i)l_1l_2c_2 + (\sum_{i=3}^{6} m_i)l_1l_3c_{23} + (\sum_{i=4}^{6} m_i)l_1l_4c_{234} + (m_5+m_6)l_1l_5c_{2345} + m_6l_1l_6c_{23456}$,
$H_{13} = H_{23} + (\sum_{i=3}^{6} m_i)l_1l_3c_{23} + (\sum_{i=4}^{6} m_i)l_1l_4c_{234} + (m_5+m_6)l_1l_5c_{2345} + m_6l_1l_6c_{23456}$,
$H_{14} = H_{24} + (\sum_{i=4}^{6} m_i)l_1l_4c_{234} + (m_5+m_6)l_1l_5c_{2345} + m_6l_1l_6c_{23456}$,
$H_{15} = H_{25} + (m_5+m_6)l_1l_5c_{2345} + m_6l_1l_6c_{23456}$,
$H_{16} = H_{26} + m_6l_1l_6c_{23456}$,
$c_{\tau 1} = c_{\tau 2} - (\sum_{i=2}^{6} m_i)l_1l_2a_2s_2 - (\sum_{i=3}^{6} m_i)l_1l_3a_3s_{23} - (\sum_{i=4}^{6} m_i)l_1l_4a_4s_{234} - (m_5 + m_6)l_1l_5a_5s_{2345} + m_6l_1l_6a_6s_{23456}$,
$g_{\tau 1} = g_{\tau 2} + (\sum_{i=1}^{6} m_i)l_1gc_1$;
$H_{21} = H_{31} + (\sum_{i=2}^{6} m_i)l_2(l_1c_2 + l_2) + (\sum_{i=3}^{6} m_i)l_2l_3c_3 + (\sum_{i=4}^{6} m_i)l_2l_4c_{34} + (m_5+m_6)l_2l_5c_{345} + m_6l_2l_6c_{3456}$,
$H_{22} = H_{32} + (\sum_{i=2}^{6} m_i)l_2(l_2) + (\sum_{i=3}^{6} m_i)l_2l_3c_3 + (\sum_{i=4}^{6} m_i)l_2l_4c_{34} + (m_5+m_6)l_2l_5c_{345} + m_6l_2l_6c_{3456}$,
$H_{23} = H_{33} + (\sum_{i=3}^{6} m_i)l_2l_3c_3 + (\sum_{i=4}^{6} m_i)l_2l_4c_{34} + (m_5 + m_6)l_2l_5c_{345} + m_6l_2l_6c_{3456}$,
$H_{24} = H_{34} + (\sum_{i=4}^{6} m_i)l_2l_4c_{34} + (m_5 + m_6)l_2l_5c_{345} + m_6l_2l_6c_{3456}$,
$H_{25} = H_{35} + (m_5+m_6)l_2l_5c_{345} + m_6l_2l_6c_{3456}$,
$H_{26} = H_{36} + m_6l_2l_6c_{3456}$,
$c_{\tau 2} = c_{\tau 3} + (\sum_{i=2}^{6} m_i)l_2(l_1a_1s_2) - (\sum_{i=3}^{6} m_i)l_2l_3a_3s_3 - (\sum_{i=4}^{6} m_i)l_2l_4a_4s_{34} - (m_5+m_6)l_2l_5a_5s_{345} + m_6l_2l_6a_6s_{3456}$,
$g_{\tau 2} = g_{\tau 3} + (\sum_{i=2}^{6} m_i)l_2gc_{12}$;
$H_{31} = H_{41} + (\sum_{i=3}^{6} m_i)l_3(l_1c_{23} + l_2c_3 + l_3) + (\sum_{i=4}^{6} m_i)l_3l_4c_4 + (m_5+m_6)l_3l_5c_{45} + m_6l_3l_6c_{456}$,
$H_{32} = H_{42} + (\sum_{i=3}^{6} m_i)l_3(l_2c_3 + l_3) + (\sum_{i=4}^{6} m_i)l_3l_4c_4 + (m_5+m_6)l_3l_5c_{45} + m_6l_3l_6c_{456}$,
$H_{33} = H_{43} + (\sum_{i=3}^{6} m_i)l_3(l_3) + (\sum_{i=4}^{6} m_i)l_3l_4c_4 + (m_5 + m_6)l_3l_5c_{45} + m_6l_3l_6c_{456}$,
$H_{34} = H_{44} + (\sum_{i=4}^{6} m_i)l_3l_4c_4 + (m_5 + m_6)l_3l_5c_{45} + m_6l_3l_6c_{456}$,
$H_{35} = H_{45} + (m_5+m_6)l_3l_5c_{45} + m_6l_3l_6c_{456}$,
$H_{36} = H_{46} + m_6l_3l_6c_{456}$,
$c_{\tau 3} = c_{\tau 4} + (\sum_{i=3}^{6} m_i)l_3(l_1a_1s_{23} + l_2a_2s_3) - (\sum_{i=4}^{6} m_i)l_3l_4a_4s_4 - (m_5+m_6)l_3l_5a_5s_{45} + m_6l_3l_6a_6s_{456}$,
$g_{\tau 3} = g_{\tau 4} + (\sum_{i=3}^{6} m_i)l_3gc_{123}$;
$H_{41} = H_{51} + (m_4+m_5+m_6)l_4(l_1c_{234} + l_2c_{34} + l_3c_4 + l_4) + (m_5+m_6)l_4l_5c_5 + m_6l_4l_6c_{56}$,
$H_{42} = H_{52} + (m_4+m_5+m_6)l_4(l_2c_{34} + l_3c_4 + l_4) + (m_5 + m_6)l_4l_5c_5 + m_6l_4l_6c_{56}$,
$H_{43} = H_{53} + (m_4 + m_5 + m_6)l_4(l_3c_4 + l_4) + (m_5 + m_6)l_4l_5c_5 + m_6l_4l_6c_{56}$,
$H_{44} = H_{54} + (m_4+m_5+m_6)l_4(l_4) + (m_5+m_6)l_4l_5c_5 + m_6l_4l_6c_{56}$,
$H_{45} = H_{55} + (m_5+m_6)l_4l_5c_5 + m_6l_4l_6c_{56}$,
$H_{46} = H_{56} + m_6l_4l_6c_{56}$,
$c_{\tau 4} = c_{\tau 5} + (m_4+m_5+m_6)l_4(l_1a_1s_{234} + l_2a_2s_{34} + l_3a_3s_4) - (m_5+m_6)l_4l_5a_5s_5 + m_6l_4l_6a_6s_{56}$,
$g_{\tau 4} = g_{\tau 5} + (m_4+m_5+m_6)l_4gc_{1234}$;
$H_{51} = H_{61} + (m_5+m_6)l_5(l_1c_{2345} + l_2c_{345} + l_3c_{45} + l_4c_5 + l_5) + m_6l_5l_6c_6$,
$H_{52} = H_{62} + (m_5+m_6)l_5(l_2c_{345} + l_3c_{45} + l_4c_5 + l_5) + m_6l_5l_6c_6$,
$H_{53} = H_{63} + (m_5+m_6)l_5(l_3c_{45} + l_4c_5 + l_5) + m_6l_5l_6c_6$,
$H_{54} = H_{64} + (m_5+m_6)l_5(l_4c_5 + l_5) + m_6l_5l_6c_6$,
$H_{55} = H_{65} + (m_5+m_6)l_5(l_5) + m_6l_5l_6c_6$,
$H_{56} = H_{66} + m_6l_5l_6c_6$,
$c_{\tau 5} = c_{\tau 6} + (m_5+m_6)l_5(l_1a_1s_{2345} + l_2a_2s_{345} + l_3a_3s_{45} + l_4a_4s_5) - m_5l_5l_6a_6s_6$,
$g_{\tau 5} = g_{\tau 6} + (m_5+m_6)l_5gc_{12345}$;
$H_{61} = m_6l_6(l_1c_{23456} + l_2c_{3456} + l_3c_{456} + l_4c_{56} + l_5c_6 + l_6)$,
$H_{62} = m_6l_6(l_2c_{3456} + l_3c_{456} + l_4c_{56} + l_5c_6 + l_6)$,
$H_{63} = m_6l_6(l_3c_{456} + l_4c_{56} + l_5c_6 + l_6)$,
$H_{64} = m_6l_6(l_4c_{56} + l_5c_6 + l_6)$,
$H_{65} = m_6l_6(l_5c_6 + l_6)$,
$H_{66} = m_6l_6(l_6)$,
$c_{\tau 6} = m_6l_6(l_6)(l_1a_1s_{23456} + l_2a_2s_{3456} + l_3a_3s_{456} + l_4a_5s_{56} + l_5a_5s_6)$,
$g_{\tau 6} = m_6l_6gc_{123456}$;
in which, m_i denotes the mass of the ith link of the manipulator; l_i denotes the length of the ith link; and $a_i = (\sum_{j=1}^{i} \dot{\theta}_j)^2$ and $b_i = \sum_{j=1}^{i} \ddot{\theta}_j$, with $i = 1, 2, \cdots, 6$. Besides, g denotes the gravitational acceleration. Additionally, $c_i = \cos(\theta_i)$, $s_i = \sin(\theta_i)$, $c_{ijk\cdots} = \cos(\theta_i + \theta_j + \theta_k + \theta\ldots)$ and $s_{ijk\cdots} = \sin(\theta_i + \theta_j + \theta_k + \theta\ldots)$. Specifically speaking, for the manipulator used in this paper, $m_1 = 7.887$ kg, $m_2 = 5.730$ kg, $m_3 = 3.198$ kg, $m_4 = 3.020$ kg, $m_5 = 2.773$ kg, $m_6 = 0.337$ kg, $l_1 = 0.301$ m, $l_2 = 0.290$ m, $l_3 = 0.230$ m, $l_4 = 0.225$ m, $l_5 = 0.214$ m and $l_6 = 0.103$ m.

References

[1] Y. Zhang and Z. Zhang, *Repetitive Motion Planning and Control of Redundant Robot Manipulators*. New York: Springer-Verlag, 2013.

[2] J. J. Craig, *Introduction to Robotics: Mechanics and Control*. New Jersey, USA: Pearson Education, 2008.

[3] S. K. Saha, *Introduction to Robotics*. Noida, UP, India: Tata McGraw-Hill Education, 2008.
